# Genetic correlation between circulating metabolites and chalazion: a two-sample Mendelian randomization study

**DOI:** 10.3389/fmolb.2024.1368669

**Published:** 2024-03-21

**Authors:** Xin Zhang, Yuying Cai, Yaping Jiang, Wei Du, Weishu An, Qiangqiang Fu, Yihui Chen

**Affiliations:** ^1^ Department of Ophthalmology, Yangpu Hospital, School of Medicine, Tongji University, Shanghai, China; ^2^ Department of Oncology, Shanghai Ninth People’s Hospital, Shanghai Jiao Tong University School of Medicine, Shanghai, China; ^3^ Department of General Practice, Clinical Research Center for General Practice, Yangpu Hospital, School of Medicine, Tongji University, Shanghai, China

**Keywords:** eyelid lesion, chalazion, circulating metabolites, alanine, fatty acids, Mendelian randomization

## Abstract

**Background:** Lipid metabolism disorders were observationally associated with chalazion, but the causality of the related circulating metabolites on chalazion remained unknown. Here, we investigated the potential causal relationship between circulating metabolites and chalazion using two-sample Mendelian randomization (MR) analysis.

**Methods:** For the primary analysis, 249 metabolic biomarkers were obtained from the UK Biobank, and 123 circulating metabolites were obtained from the publication by Kuttunen et al. for the secondary analysis. Chalazion summary data were obtained from the FinnGen database. Inverse variance weighted (IVW) is the main MR analysis method, and the MR assumptions were evaluated in sensitivity and colocalization analyses.

**Results:** Two MR analyses results showed that the common metabolite, alanine, exhibited a genetic protective effect against chalazion (primary analysis: odds ratio [OR] = 0.680; 95% confidence interval [CI], 0.507–0.912; *p* = 0.010; secondary analysis: OR = 0.578; 95% CI, 0.439–0.759; *p* = 0.00008). The robustness of the findings was supported by heterogeneity and horizontal pleiotropy analysis. Two colocalization analyses showed that alanine did not share a region of genetic variation with chalazion (primary analysis: PPH_4_ = 1.95%; secondary analysis: PPH_4_ = 25.3%). Moreover, previous studies have suggested that an increase in the degree of unsaturation is associated with an elevated risk of chalazion (OR = 1.216; 95% CI, 1.055–1.401; *p* = 0.007), with omega-3 fatty acids (OR = 1.204; 95% CI, 1.054–1.377; *p* = 0.006) appearing to be the major contributing factor, as opposed to omega-6 fatty acids (OR = 0.850; 95% CI, 0.735–0.982; *p* = 0.027).

**Conclusion:** This study suggests that alanine and several unsaturated fatty acids are candidate molecules for mechanistic exploration and drug target selection in chalazion.

## Introduction

Chalazion is a benign eyelid lesion and manifests as a chronic lipogranulomatous inflammatory response secondary to the degeneration of endogenous lipid secretion from the meibomian or Zeis glands (Knop and Knop, 2009). Infectious factors, such as blepharitis, viral infection, and ocular demodex infestation (Liang et al., 2014; Kim et al., 2023), along with non-infectious factors [such as rosacea, anxiety, smoking, nutritional deficiencies, and irritable bowel syndrome (IBS)], may increase chalazion risk (Nemet et al., 2011; Patel et al., 2022).

Studies have shown that the ratio of cholesterol to cholesteryl esters is higher in chalazion lipids than in normal meibum, probably because elevated cholesterol levels influence the chemotaxis of inflammatory cells into the meibomian gland, obstructing the gland and leading to excess lipid infiltration of the surrounding tissue (Melo et al., 2011; Wojtowicz et al., 2014). Notably, a study showed that oral statins reduced chalazion risk, indicating its linkage to inadequate lipid metabolism (Feng et al., 2022). Apart from disorders of lipid metabolism, deficiencies in circulating nutrients such as amino acids, vitamins, and thyroxine can also contribute to chalazion risk (Malekahmadi et al., 2017; Cheng et al., 2022; Ilhan, 2022).

However, most studies on circulating metabolites for chalazion have been observational and susceptible to potential confounders and reverse causality (Lawlor et al., 2008). Mendelian randomization (MR) is a method that can evaluate the causal relationship between exposures and outcomes by employing a genetic variant as an instrumental variable for exposure. This approach helps to mitigate the confounding effects inherent in observational studies (Davey Smith and Ebrahim, 2005; Yuan et al., 2021). We used a two-sample MR to investigate the causal relationship between circulating metabolites and chalazion risk, and other factors for chalazion, including inflammation of the eyelid, conjunctivitis, anxiety/panic attacks, alcohol consumption, body mass index (BMI), current tobacco smoking, and IBS were analyzed to assess the effect of confounders.

## Materials and methods

### Study population and design

**FIGURE 1:**
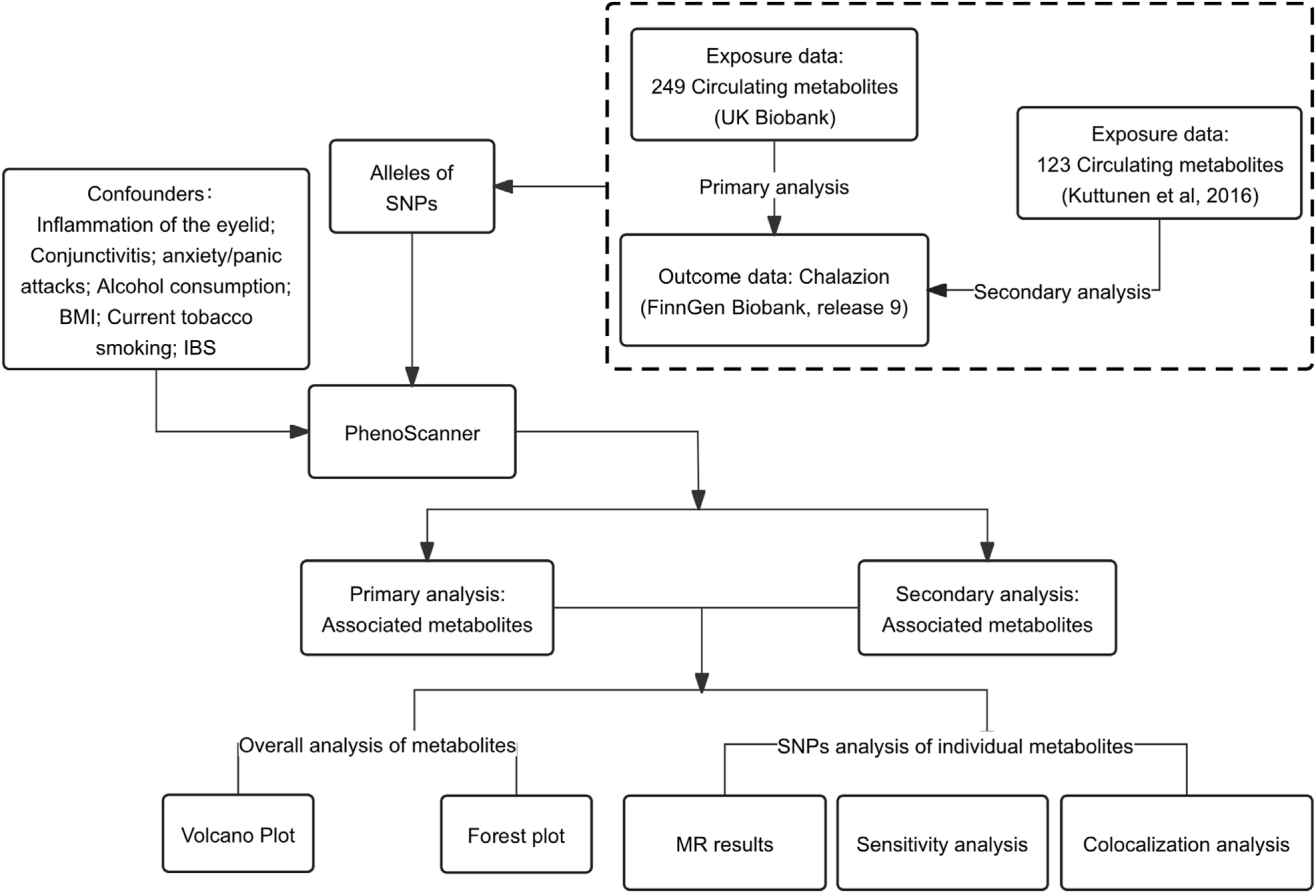
Flowchart of the present Mendelian randomization analysis. Abbreviations: SNPs, single nucleotide polymorphisms; MR, Mendelian randomization; IBS, irritable bowel syndrome; BMI, body mass index.

We conducted a two-sample MR analysis to determine the causal effect of circulating metabolites on chalazion. Genome-wide association study (GWAS) summary level data for circulating metabolites were obtained from two different datasets, and chalazion summary data were obtained from the FinnGen database. The robustness of these causal relationships was assessed by sensitivity and colocalization analyses. Only individuals of European origin were included in the analysis. All included studies received informed consent and local ethics committee approval. An overview of the study design is shown in Figure 1.

### Sources of data included in the analysis

Summary-level GWAS datasets of 249 metabolic biomarkers for the primary analysis were derived from the UK Biobank and measured using the Nightingale Health Metabolic Biomarkers Phase 1 release study. This study included 115,078 randomly selected participants from the UK Biobank cohort, and analysed a total of 249 metabolites, with 168 metabolites measured in absolute concentrations (mmol/L) and 81 measurements presented in ratios (https://biobank.ndph.ox.ac.uk/ukb/label.cgi?id=220) (Mi et al., 2022). Summary-level datasets of 123 circulating metabolites used for secondary analyses were obtained from a previous publication by Kettunen et al. (2016), including 24,925 individuals of European ancestry. Summary-level statistics for chalazion were obtained from the FinnGen database (Release 9), which included 348,073 individuals of European ancestry (case: 3,389; control: 344,684). Confounders included eyelid inflammation, conjunctivitis, anxiety/panic attacks, alcohol consumption, BMI, smoking, and IBS, which were obtained from the IEU OpenGWAS project (https://gwas.mrcieu.ac.uk/datasets/) (Supplementary Table S1).

### Selection of genetic instrumental variables

SNPs associated with circulating metabolites were selected according to a significance threshold (*p* < 5 × 10^−8^) and a reference panel (with a window size of 10,000 kb and *r* ^2^ < 0.001) to estimate the linkage disequilibrium between SNPs and select independent genetic variants (Levin et al., 2020). The F-statistic was calculated to test for weak instruments (F-statistics < 10) as previously described (Burgess et al., 2011; Burgess and Thompson, 2011). Instrumental variables (IVs) for MR studies need to fulfil three hypotheses: 1) IVs should be strongly correlated with exposure; 2) IVs should not be associated with any confounders; and 3) IVs affect outcomes only through exposure and not through any other alternative pathways (Palmer et al., 2012).

### Colocalization analysis

The presence of shared causal variants between exposure and outcome was analysed using GWAS-GWAS colocalization, with a reference panel (GWAS gene window default of 50 kb, *p* < 5 × 10^−8^) (Venkateswaran et al., 2018). Colocalization analyses were performed using the “coloc” package, and a Bayesian framework was used to generate posterior probabilities for five mutually exclusive hypotheses about causal variance sharing between two traits: (H_0_–H_4_): H_0_: the locus is not associated with circulating metabolites and chalazion; H_1_: the locus is associated with circulating metabolites, but not with chalazion; H_2_: the locus is associated with chalazion, but not with circulating metabolites; H_3_: the locus is associated with circulating metabolites and chalazion (two independent SNPs) and H_4_: the locus is associated with circulating metabolites and chalazion (one shared SNP) (Giambartolomei et al., 2014; Venkateswaran et al., 2018). A posterior probability (PP) of H_4_ (PPH_4_) > 0.8 was identified as evidence in favour of colocalization. The “locuscomparer” package was used for visualization of colocalization (Liu et al., 2019).

### Statistical analysis

Causal associations between each metabolite and chalazion were determined using IVW methods. The IVW estimate is the mean of ratio estimates from two or more instruments, based on the assumption that all SNPs are valid instruments or that the overall bias is zero (Burgess et al., 2013). MR Egger regression, weighted median, and weighted mode methods were used to relax the IVW assumptions (Burgess et al., 2017a; Burgess et al., 2019). For single genetic instruments, causal effects were estimated by the wald ratio test (Burgess et al., 2017b). Phenoscanner (http://www.phenoscanner.medschl.cam.ac.uk/) was used to exclude the SNPs associated with confounders (Staley et al., 2016). Sensitivity analyses were performed to assess the validity and robustness of the MR assumptions, including Cochran’s Q to test for heterogeneity among IVs of metabolites (Burgess et al., 2013), MR-Egger regression for horizontal pleiotropy assessment (Bowden et al., 2015), and leave-one-out test. If heterogeneity was present, the multiplicative random effects model was chosen (Burgess et al., 2019). MR-Steiger was used to filter out SNPs suggesting opposite causal directions (Hemani et al., 2017).

We chose the Bonferroni method for multiple testing; associations with *p*-values below 0.0002 (0.05/249) in the primary analysis and 0.0004 (0.05/121) in the secondary analysis were considered strong evidence of an association, and associations with *p*-values between 0.0002 and 0.05 in the primary analysis, and between 0.0004 and 0.05 in the secondary analysis were considered suggestive. All analyses were performed on the R platform (version 4.1.0) using the “TwoSampleMR,” “Coloc,” and “forestploter” packages.

## Results

### Association of relevant confounders with chalazion

We first estimated the causal relationship of confounders, including inflammation of the eyelid, conjunctivitis, anxiety/panic attacks, alcohol consumption, BMI, current tobacco smoking, and IBS, with chalazion risk, using a two-sample MR method. The results showed that BMI [OR = 0.970; 95% CI, 0.826–1.138; P_(IVW)_ = 0.709], Current tobacco smoking [OR = 0.724; 95% CI, 0.214–2.454; P_(IVW)_ = 0.604], Alcohol consumption [OR = 1.334; 95% CI, 0.099–17.99; P_(IVW)_ = 0.828], IBD [OR = 2.49E-06; 95% CI, 9.06E-24-6.81704E+11; P_(Wald ratio)_ = 0.529], and conjunctivitis [OR = 0.945; 95% CI, 0.495–1.805; P_(Wald ratio)_ = 0.863] were not association with chalazion risk, and the remaining confounders failed to establish associations with chalazion due to the absence of SNPs (Supplementary Table S2).

### Association of metabolites on chalazion

**FIGURE 2:**
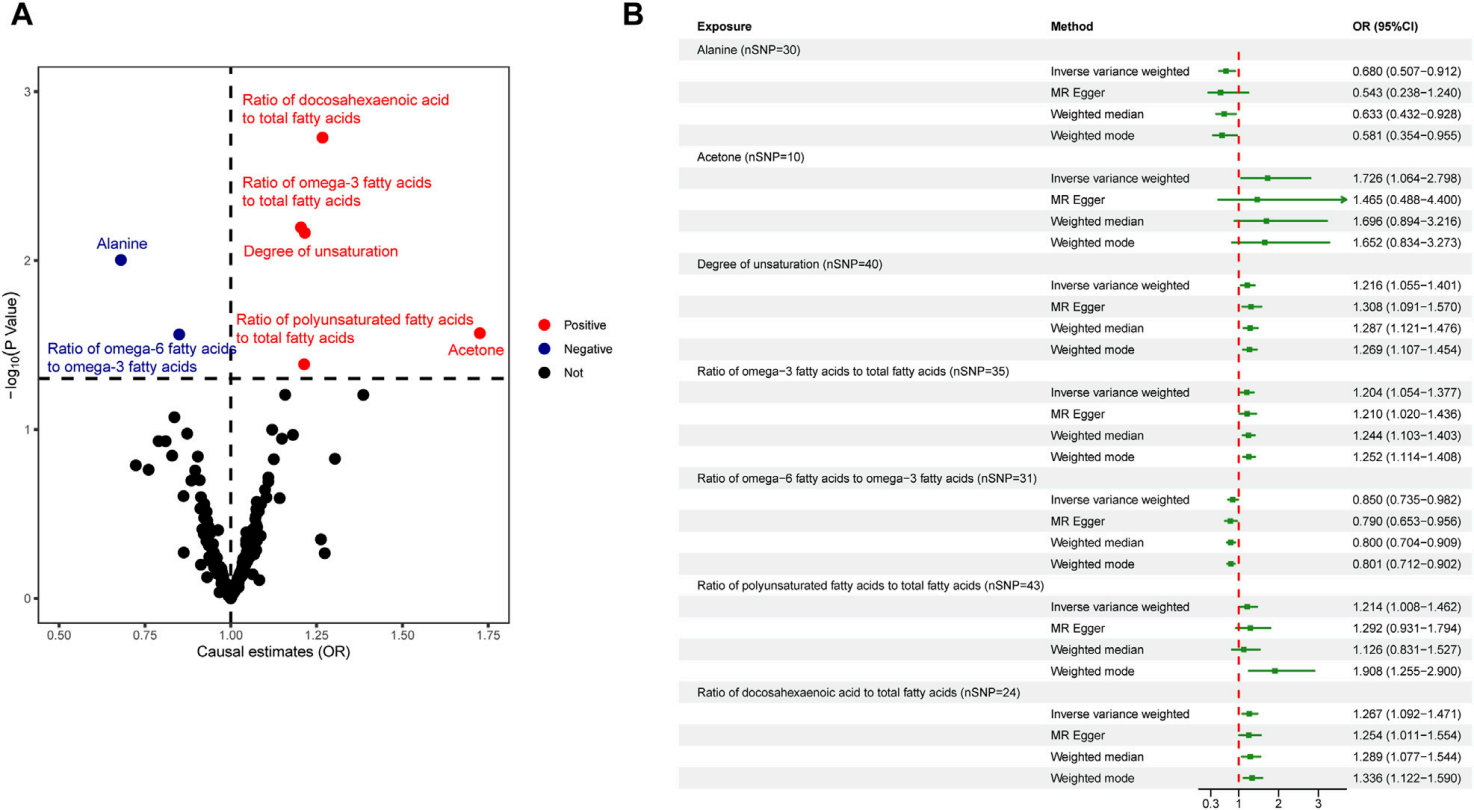
Causal relationship between 249 metabolites and chalazion. **(A)** Volcano plot analysis of Mendelian randomization results. Red plots indicate OR > 1, which is positively associated with the risk of developing chalazion, and blue plots indicate OR < 1, which is negatively associated with the risk of developing chalazion. **(B)** Forest plot analysis of Mendelian randomization results. Abbreviations: OR, odds ratio.

**FIGURE 3:**
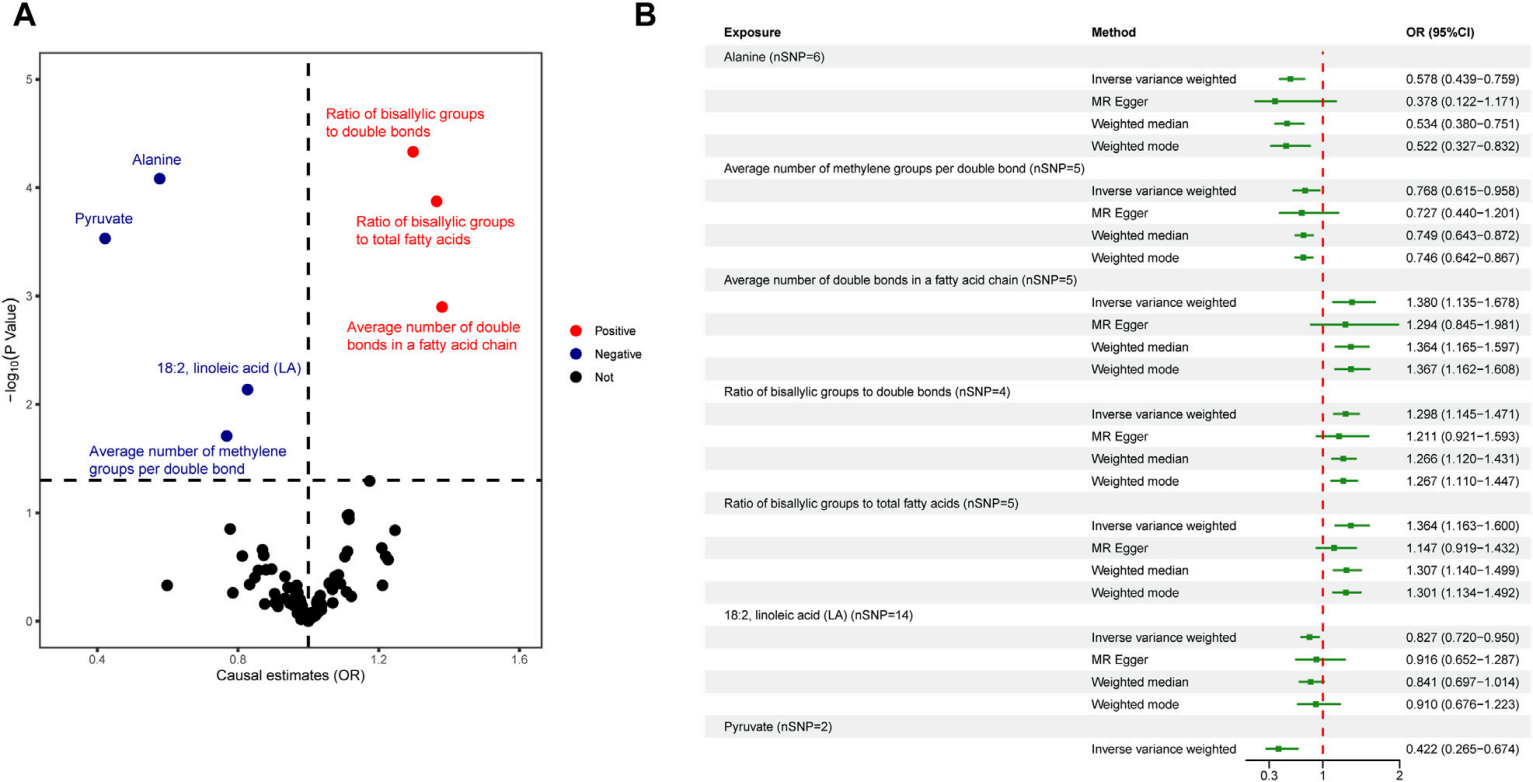
Causal relationship between 123 metabolites and chalazion. **(A)** Volcano plot analysis of Mendelian randomization results. Red plots indicate OR > 1, which is positively associated with the risk of developing chalazion, and blue plots indicate OR < 1, which is negatively associated with the risk of developing chalazion. **(B)** Forest plot analysis of Mendelian randomization results. Abbreviations: OR, odds ratio.

In the primary analysis, we screened 249 circulating metabolites with a total of 13,275 SNPs when the screening threshold was (*r* ^2^ < 0.001, kb = 10,000, *p* < 5 × 10^−8^). In the secondary analysis, we screened 123 circulating metabolites with a total of 1,375 SNPs when the screening threshold was (*r* ^2^ < 0.001, kb = 10,000, *p* < 5 × 10^−8^), among them, two metabolites glycerol (met-c-861) and urea (met-c-939), had no SNP extracted. All IVs in the current analysis were robust instruments, with the range of F-statistics was 23-16414 (Supplementary Tables S3, S4). We used a two-sample MR to estimate the causal effect of 249 metabolites on chalazion in the primary analysis and 121 circulating metabolic biomarkers risk in the secondary analysis (Supplementary Tables S5, S6). Based on the IVW method, seven of the 249 metabolites were found to be suggestively associated with chalazion, with five unsaturation-associated biomarkers showing an association with a high risk of chalazion and two unsaturation-associated biomarkers showing a causal effect with a low risk of a chalazion (Figure 2A). There were as follows: alanine (OR = 0.680; 95% CI, 0.507–0.912; *p* = 0.010), acetone (OR = 1.726; 95% CI, 1.064–2.798; *p* = 0.027), degree of unsaturation (OR = 1.216; 95% CI, 1.055–1.401; *p* = 0.007), ratio of omega-3 fatty acids to total fatty acids (OR = 1.204; 95% CI, 1.054–1.377; *p* = 0.006), ratio of omega-6 to omega-3 fatty acids (OR = 0.850; 95% CI, 0.735–0.982; *p* = 0.027), ratio of polyunsaturated fatty acids to total fatty acids (OR = 1.214; 95% CI, 1.008–1.462; *p* = 0.041), and ratio of docosahexaenoic acid to total fatty acids (OR = 1.267; 95% CI, 1.092–1.471; *p* = 0.002) (Figure 2B; Table 1). In the secondary analysis, four of the 121 metabolites were found to be significantly associated with chalazion and three were suggestively associated with chalazion. Of these, three unsaturation-associated biomarkers significantly increased the risk of chalazion, and four unsaturation-associated biomarkers showed a negative correlation with chalazion susceptibility (Figure 3A). They were alanine (OR = 0.578; 95% CI, 0.439–0.759; *p* = 0.00008), the average number of methylene groups per double bond (OR = 0.768; 95% CI, 0.615–0.958; *p* = 0.020), the average number of double bonds in a fatty acid chain (OR = 1.380; 95% CI, 1.135–1.678; *p* = 0.001), ratio of bisallylic groups to double bonds (1.298 [95% CI, 1.145–1.471], *p* = 0.00005), ratio of bisallylic groups to total fatty acids (OR = 1.364; 95% CI, 1.163–1.600; *p* = 0.00013), 18:2, linoleic acid (LA) (OR = 0.827; 95% CI, 0.720–0.950; *p* = 0.007), and pyruvate (OR = 0.422; 95% CI, 0.265–0.674; *p* = 0.00029) (Figure 3B; Table 2). MR results of individual SNPs for all metabolites were shown in Supplementary Tables S7, S8.

**TABLE 1:** MR analysis results of seven of 249 metabolites with a causal relationship with chalazion.

Exposure	Category	Method	nSNP	Beta	SE	*p*-value	OR (95% CI)
Alanine	Amino acid	IVW	30	−0.386	0.150	0.010	0.680 (0.507–0.912)
		MR Egger	30	−0.610	0.421	0.158	0.543 (0.238–1.240)
		Weighted median	30	−0.457	0.195	0.019	0.633 (0.432–0.928)
		Weighted mode	30	−0.543	0.253	0.041	0.581 (0.354–0.955)
Acetone	Metabolite	IVW	10	0.546	0.247	0.027	1.726 (1.064–2.798)
		MR Egger	10	0.382	0.561	0.515	1.465 (0.488–4.400)
		Weighted median	10	0.528	0.326	0.106	1.696 (0.894–3.216)
		Weighted mode	10	0.502	0.349	0.184	1.652 (0.834–3.273)
Degree of unsaturation	Fatty acid	IVW_mre_	40	0.195	0.072	0.007	1.216 (1.055–1.401)
		MR Egger	40	0.269	0.093	0.006	1.308 (1.091–1.570)
		Weighted median	40	0.252	0.070	0.000	1.287 (1.121–1.476)
		Weighted mode	40	0.238	0.070	0.001	1.269 (1.107–1.454)
Ratio of omega-3 fatty acids to total fatty acids	Fatty acid	IVW_mre_	35	0.186	0.068	0.006	1.204 (1.054–1.377)
		MR Egger	35	0.191	0.087	0.036	1.210 (1.020–1.436)
		Weighted median	35	0.218	0.061	0.000	1.244 (1.103–1.403)
		Weighted mode	35	0.225	0.060	0.001	1.252 (1.114–1.408)
Ratio of omega-6 fatty acids to omega-3 fatty acids	Fatty acid	IVW_mre_	31	−0.163	0.074	0.027	0.850 (0.735–0.982)
		MR Egger	31	−0.235	0.097	0.022	0.790 (0.653–0.956)
		Weighted median	31	−0.223	0.065	0.001	0.800 (0.704–0.909)
		Weighted mode	31	−0.221	0.060	0.001	0.801 (0.712–0.902)
Ratio of polyunsaturated fatty acids to total fatty acids	Fatty acid	IVW	43	0.194	0.095	0.041	1.214 (1.008–1.462)
		MR Egger	43	0.256	0.167	0.133	1.292 (0.931–1.794)
		Weighted median	43	0.119	0.155	0.444	1.126 (0.831–1.527)
		Weighted mode	43	0.646	0.214	0.004	1.908 (1.255–2.900)
Ratio of docosahexaenoic acid to total fatty acids	Fatty acid	IVW	24	0.237	0.076	0.002	1.267 (1.092–1.471)
		MR Egger	24	0.226	0.110	0.051	1.254 (1.011–1.554)
		Weighted median	24	0.254	0.092	0.006	1.289 (1.077–1.544)
		Weighted mode	24	0.289	0.089	0.004	1.336 (1.122–1.590)

Abbreviations: IVW, inverse variance weighted; IVW_mre_, inverse variance weighted (multiplicative random effects); SNPs, single nucleotide polymorphisms; SE, standard error; OR, odds ratio; CI, confidence interval.

**TABLE 2:** MR analysis results of seven of 123 metabolites with a causal relationship with chalazion.

Exposure	Category	Method	nSNP	Beta	SE	*p*-value	OR (95% CI)
Alanine	Amino acid	IVW	6	−0.549	0.139	0.000	0.578 (0.439–0.759)
		MR Egger	6	−0.974	0.578	0.167	0.378 (0.122–1.171)
		Weighted median	6	−0.626	0.174	0.000	0.534 (0.380–0.751)
		Weighted mode	6	−0.651	0.238	0.041	0.522 (0.327–0.832)
Average number of methylene groups per double bond	Metabolites	IVW_mre_	5	−0.264	0.113	0.020	0.768 (0.615–0.958)
		MR Egger	5	−0.319	0.256	0.301	0.727 (0.440–1.201)
		Weighted median	5	−0.289	0.078	0.000	0.749 (0.643–0.872)
		Weighted mode	5	−0.293	0.077	0.019	0.746 (0.642–0.867)
Average number of double bonds in a fatty acid chain	Fatty acid	IVW	5	0.322	0.100	0.001	1.380 (1.135–1.678)
		MR Egger	5	0.258	0.217	0.321	1.294 (0.845–1.981)
		Weighted median	5	0.310	0.080	0.000	1.364 (1.165–1.597)
		Weighted mode	5	0.313	0.083	0.019	1.367 (1.162–1.608)
Ratio of bisallylic groups to double bonds	Metabolites	IVW	4	0.261	0.064	0.000	1.298 (1.145–1.471)
		MR Egger	4	0.192	0.140	0.304	1.211 (0.921–1.593)
		Weighted median	4	0.236	0.062	0.000	1.266 (1.120–1.431)
		Weighted mode	4	0.237	0.068	0.040	1.267 (1.110–1.447)
Ratio of bisallylic groups to total fatty acids	Fatty acid	IVW	5	0.311	0.081	0.000	1.364 (1.163–1.600)
		MR Egger	5	0.137	0.113	0.313	1.147 (0.919–1.432)
		Weighted median	5	0.268	0.070	0.000	1.307 (1.140–1.499)
		Weighted mode	5	0.263	0.070	0.020	1.301 (1.134–1.492)
18:2, linoleic acid (LA)	Fatty acid	IVW	14	−0.190	0.071	0.007	0.827 (0.720–0.950)
		MR Egger	14	−0.088	0.174	0.622	0.916 (0.652–1.287)
		Weighted median	14	−0.174	0.096	0.070	0.841 (0.697–1.014)
		Weighted mode	14	−0.095	0.151	0.542	0.910 (0.676–1.223)
Pyruvate	Amino acid	IVW	2	−0.862	0.238	0.000	0.422 (0.265–0.674)

Abbreviations: IVW, inverse variance weighted; IVWmre, inverse variance weighted (multiplicative random effects); SNPs, single nucleotide polymorphisms; SE, standard error; OR, odds ratio; CI, confidence interval.

### Sensitivity analysis

In the sensitivity analysis of all the differential metabolites, heterogeneity (IVW method) was present only for unsaturation (*p* = 0.045), ratio of omega-6 to omega-3 fatty acids (*p* = 0.008), ratio of omega-3 fatty acids to total fatty acids (*p* = 0.018) in 249 metabolites (Supplementary Table S9), and average number of methylene groups per double bond (*p* = 0.016) in 123 metabolites (Supplementary Table S10), and we adopted a MR analysis with multiplicative random effects model. The horizontal pleiotropy analysis showed that there was no direct association between IVs and chalazion, and MR Steiger test results showed overall SNPs affected outcomes only by exposure and not by other pathways (Supplementary Tables S11, S12), suggesting that the results were relatively robust. Plots of the leave-one-out test showed that regardless of which SNP was excluded, it did not have any effect on the MR results for alanine, ratio of bisallylic groups to double bonds, and ratio of bisallylic groups to total fatty acids (All lines were on one side of 0). However, for other unsaturated fatty acids, there is a potentially influential SNP driving the causal link between these metabolites and chalazion. Therefore, we need to interpret this result with caution (Supplementary Figure S1 **)**.

### Colocalization analysis

Two colocalization analyses showed that there was no shared genetic variation region between alanine and chalazion (1.95% for PPH4 in the primary analysis and 25.3% in the secondary analysis), suggesting that the genetic variant SNPs were reducing chalazion risk through their impact on alanine. Shared regions of genetic variation were not observed for the differential metabolites in the primary analysis. In the secondary analysis, we identified two metabolites: the ratio of bisallylic groups to double bonds (PPH_4_ = 0.817) and 18:2, linoleic acid (LA) (PPH_4_ = 0.870), which shared one SNP with chalazion (rs174555), corresponding to the nearest genes FADS1 and FADS2 (Table 3; Supplementary Figure S2).

**TABLE 3:** Summary of Mendelian randomization sensitivity and gene colocalisation analysis.

Exposure	Outcome	Method	P_heterogeneity_	P_pleiotropy_	PPH_3_	PPH_4_	nSNP in colocalisation
Primary analysis							
Alanine	Chalazion	IVW	0.119	0.573	0.012	0.019	221
Acetone	Chalazion	IVW	0.751	0.754	0.014	0.038	235
Degree of unsaturation	Chalazion	IVW	0.045	0.221	0.014	0.028	147
Ratio of omega-3 fatty acids to total fatty acids	Chalazion	IVW	0.018	0.930	0.013	0.048	142
Ratio of omega-6 fatty acids to omega-3 fatty acids	Chalazion	IVW	0.008	0.265	0.014	0.025	144
Ratio of polyunsaturated fatty acids to total fatty acids	Chalazion	IVW	0.337	0.650	0.156	0.722	257
Ratio of docosahexaenoic acid to total fatty acids	Chalazion	IVW	0.441	0.890	0.013	0.516	187
Secondary analysis							
Alanine	Chalazion	IVW	0.878	0.491	0.012	0.254	193
Average number of methylene groups per double bond	Chalazion	IVW	0.349	0.819	0.116	0.792	222
Average number of double bonds in a fatty acid chain	Chalazion	IVW	0.162	0.752	0.117	0.790	224
Ratio of bisallylic groups to double bonds	Chalazion	IVW	0.032	0.622	0.099	0.817	201
Ratio of bisallylic groups to total fatty acids	Chalazion	IVW	0.147	0.160	0.116	0.793	222
18:2, linoleic acid (LA)	Chalazion	IVW	0.314	0.531	0.072	0.870	256
Pyruvate	Chalazion	IVW	0.870	—	0.011	0.297	194

Abbreviations: IVW, inverse variance weighted; SNPs, single nucleotide polymorphisms; PPH_3_, posterior probability (PP) of H_3_; PPH_4_, posterior probability (PP) of H_4_.

## Discussion

In this study, we conducted an MR analysis to explore the causal relationship between circulating metabolites and chalazion. The primary analysis results showed a negative causal association between the metabolite alanine and chalazion risk, which was similarly verified in the secondary analysis. Sensitivity analyses enhanced the reliability of our results in eliminating pleiotropy. The colocalization analyses showed that genetic variant SNPs could exclusively mitigate the risk of chalazion through their influence on alanine. Alanine is the link between glucose, the tricarboxylic acid cycle, and amino acid metabolism and can form pyruvate and glutamate via an alanine aminotransferase-catalysed reaction with α-ketoglutarate, which plays a key role in the glucose-alanine cycle between tissues and the liver (Peng et al., 1991). Alanine can shuttle between neurones and astrocytes, transport nitrogen from the glutamine amide group, exchange lactate, and facilitate metabolite exchange between neurones and astrocytes (Schousboe et al., 2003). Previous studies have found that concentrations of lactate and alanine are low in patients with type 2 diabetes mellitus, breast cancer, and cervical cancer, suggesting altered energy metabolism in these diseases (Morvan and Demidem, 2007; Mamtimin et al., 2014).

In addition to alanine, pyruvate was effective in reducing the risk of chalazion development. Pyruvate is produced from alanine in the liver by the reaction of alanine aminotransferase to produce pyruvate and regenerate glucose in the body through gluconeogenesis to provide energy (Prochownik and Wang, 2021). Pyruvate is an important product of glycolysis and can be converted to acetyl-CoA before entering the tricarboxylic acid (TCA) cycle (Han et al., 2020), and acetyl-CoA is an important intermediate metabolite in the metabolism of energy substances. Alanine and pyruvate are closely related to the metabolic pathways of alanine, aspartate and glutamate metabolism, the TCA cycle, glycolysis or gluconeogenesis, and pyruvate metabolism, which are important for the maintenance of normal physiological states of the retina (Rueda et al., 2016), optic nerve cells (Agudo-Barriuso et al., 2013), lens (Saravanan et al., 2023), and cornea (Hamuro et al., 2020). And these studies give us confidence that alanine and pyruvate may be biomarkers in the metabolic process of eye diseases.

Our study also identified some associations between unsaturated fatty acids and chalazion risk. In the primary analysis, the higher the percentage of unsaturated fatty acids, the higher the risk of chalazion. We found that the higher the ratio of omega-6 to omega-3 fatty acids, the lower the risk of chalazion, suggesting that omega-6 fatty acids protect against chalazion among unsaturated fatty acids, whereas omega-3 fatty acids and other unsaturated fatty acids may increase the risk of chalazion. In the results of the secondary analysis, the ability of 18:2, LA [an omega-6 fatty acid (Balić et al., 2020)] to reduce the risk of chalazion also supports the results of the primary analysis of the protective effect of omega-6 fatty acids on chalazion. This has been somewhat controversial in previous studies on ocular unsaturated fatty acids. Studies have shown that omega-3 fatty acids are effective in reducing inflammation associated with dry eye, improving the lipid layer of the tear film, and normalising the function of the levator palpebral and lacrimal glands (Cortina and Bazan, 2011; Georgakopoulos et al., 2017). In recent years, several studies have questioned the efficacy of omega-3 fatty acids in the treatment of dry eye and found that low concentrations of omega-6 fatty acids (not omega-3 fatty acids) are beneficial in improving tear stability in patients with dry eye (Mudgil, 2020). Similarly, in the fatty acid category of the secondary analyses, we observed that more double bonds in the fatty acids increased the risk of chalazion. A higher number and proportion of double bonds and bisallylic groups in the fatty acid chain increase the risk of chalazion formation. Unsaturated fatty acids contain more double bonds than saturated fatty acids, which is consistent with the results of our primary analysis. In summary, we found an increased risk of chalazion when fatty acids had a higher number and ratio of double bonds and bisallylic groups because omega-6 fatty acids have fewer double bonds than omega-3 fatty acids (2–4 vs. 3-6 double bonds). An increase in the number of bisallylic groups can cause oxidative stress reactions (Yamaguchi et al., 2012; Chistyakov et al., 2018). A higher number and proportion of dienophiles have been reported to increase cellular susceptibility to free radical-mediated peroxidative events, which increases the risk of chalazion (Wagner et al., 1994). The gene colocalization results highlighted the significance of targeting rs174555, which is associated with FADS1 and FADS2. FADS1 and FADS1 encode fatty acid δ-5 and δ-56 desaturases (D5D and D6D), respectively, crucial for the conversion of PUFAs (polyunsaturated fatty acids) into eicosapentaenoic acid (EPA), docosahexaenoic acid, (DHA) arachidonic acid (ARA) (Santana et al., 2022). Studies have shown that SNPs in the FADS1 and FADS2 genes [such as rs174545, rs174546, rs174548, and rs174553 (FADS1); rs1535 and rs174583 (FADS2)] can affect the bioavailability of omega-3 and omega-6 PUFAs in various tissues. They can also increase plasma levels of LA and ALA, and decrease levels of ARA, EPA, and DHA (Steer et al., 2013; de la Garza Puentes et al., 2017). Our study revealed rs174555 (FADS1, FADS2) had a two-sided effect on chalazion risk, being associated with the ratio of bisallylic groups to double bonds (1.298 [95% CI, 1.145–1.471], PPH_4_ = 0.817) and 18:2, linoleic acid (LA, 0.827 [95% CI, 0.720–0.950], PPH_4_ = 0.870) metabolism. This suggests that rs174555 (FADS1, FADS2) as a drug target related to fatty acid metabolism needs to be considered carefully for the presence of bidirectional effects.

Acetone was associated with a higher chalazion risk. It is a metabolite of fatty acids (Møller, 2020), and we hypothesised that increased fatty acid levels would increase the risk of chalazion and may be partly related to the fatty acid metabolite acetone. However, further research is required to verify these hypotheses. A higher average number of methylene groups per double bond reduces the risk of chalazion formation. Studies have shown that methylene groups can increase the lipophilicity of compounds (Møller, 2020), and in addition, methylene groups can act as methylating agents, playing a key role in DNA methylation (Botezatu et al., 2014). Additionally, methylene groups are involved in the synthesis of many amino acids and biologically active molecules as well as in metabolic processes (Stocke and Lamont, 2023).

However, it is essential to acknowledge the primary limitation of this MR analysis: First, the study was based exclusively on patients with chalazion of European origin, which limits its generalisability to other ethnic populations. Further testing of GWAS for metabolites and chalazions in other populations would facilitate larger MR analyses. Second, although we did not observe multidirectional evidence of causal associations across the MR methods, the variants used in MR may confer a risk for chalazion development through multidirectional pathways. Further MR analyses using individual-level data should be performed to assess the potential dose-response causality between individual metabolites and chalazion risk. Meanwhile, further studies are required to elucidate the potential mechanisms underlying these findings.

This study offers evidence supporting the role of alanine in providing protection against chalazion, suggesting its significance as a potential metabolite in chalazion treatment. We also suggest that the degree of unsaturation in circulating fatty acids is a prominent factor in the development of chalazion, highlighting the need for further investigation into the mechanisms of omega-3 and omega-6 fatty acids in this context.

## Data Availability

The original contributions presented in the study are included in the article/Supplementary Material, further inquiries can be directed to the corresponding authors.

## Supplementary material

SUPPLEMENTARY FIGURE S1 Leave-one-out tests for 13 metabolites on chalazion in two MR analysis (except for Pyruvate). Primary analysis **(A–G)**: **(A)** alanine; **(B)** acetone; **(C)** degree of unsaturation; **(D)** ratio of omega-3 fatty acids to total fatty acids; **(E)** ratio of omega-6 fatty acids to omega-3 fatty acids; **(F)** ratio of polyunsaturated fatty acids to total fatty acids; **(G)** ratio of docosahexaenoic acid to total fatty acids. Secondary analysis **(H–M)**: **(H)** alanine; **(I)** average number of methylene groups per double bond; **(J)** average number of double bonds in a fatty acid chain; **(K)** ratio of bisallylic groups to double bonds; **(L)** ratio of bisallylic groups to total fatty acids; **(M)** 18:2, linoleic acid (LA).

SUPPLEMENTARY FIGURE S2 Colocalization analyses for 14 metabolites on chalazion in two MR analysis. Primary analysis **(A–G)**: **(A)** alanine; **(B)** acetone; **(C)** degree of unsaturation; **(D)** ratio of omega-3 fatty acids to total fatty acids; **(E)** ratio of omega-6 fatty acids to omega-3 fatty acids; **(F)** ratio of polyunsaturated fatty acids to total fatty acids; **(G)** ratio of docosahexaenoic acid to total fatty acids. Secondary analysis **(H–N)**: **(H)** alanine; **(I)** average number of methylene groups per double bond; **(J)** average number of double bonds in a fatty acid chain; **(K)** ratio of bisallylic groups to double bonds; **(L)** ratio of bisallylic groups to total fatty acids; **(M)** 18:2, linoleic acid (LA); **(N)** pyruvate.
