# Supplementary figures and images for: Genetic correlation between circulating metabolites and chalazion: a two-sample Mendelian randomization study
Source: Front Mol Biosci. 2024 Mar 21;11:1368669. doi: 10.3389/fmolb.2024.1368669 (PMC10991826; doi:10.3389/fmolb.2024.1368669)

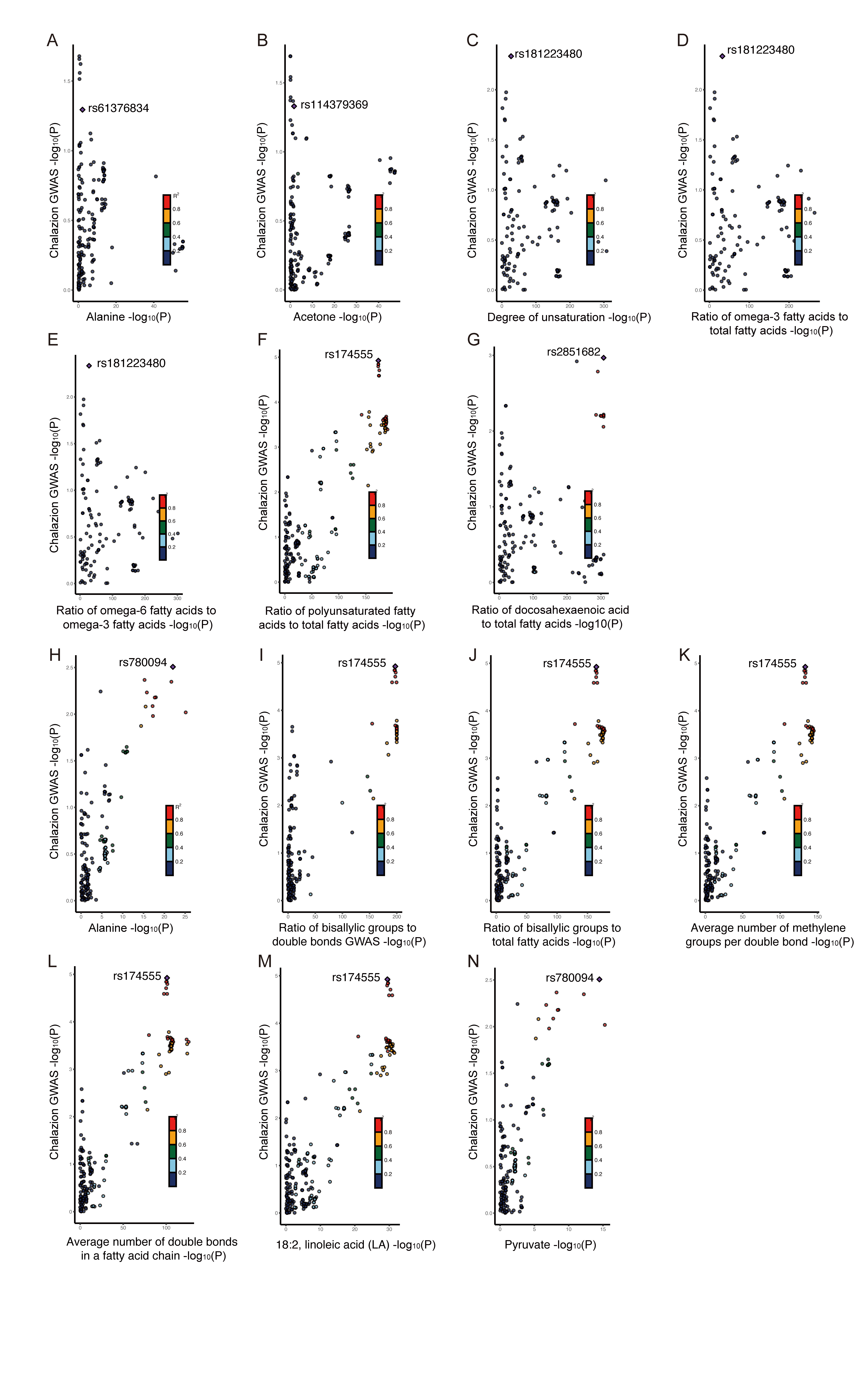

Supplement: Supplementary file 1 [file Image2.TIF]

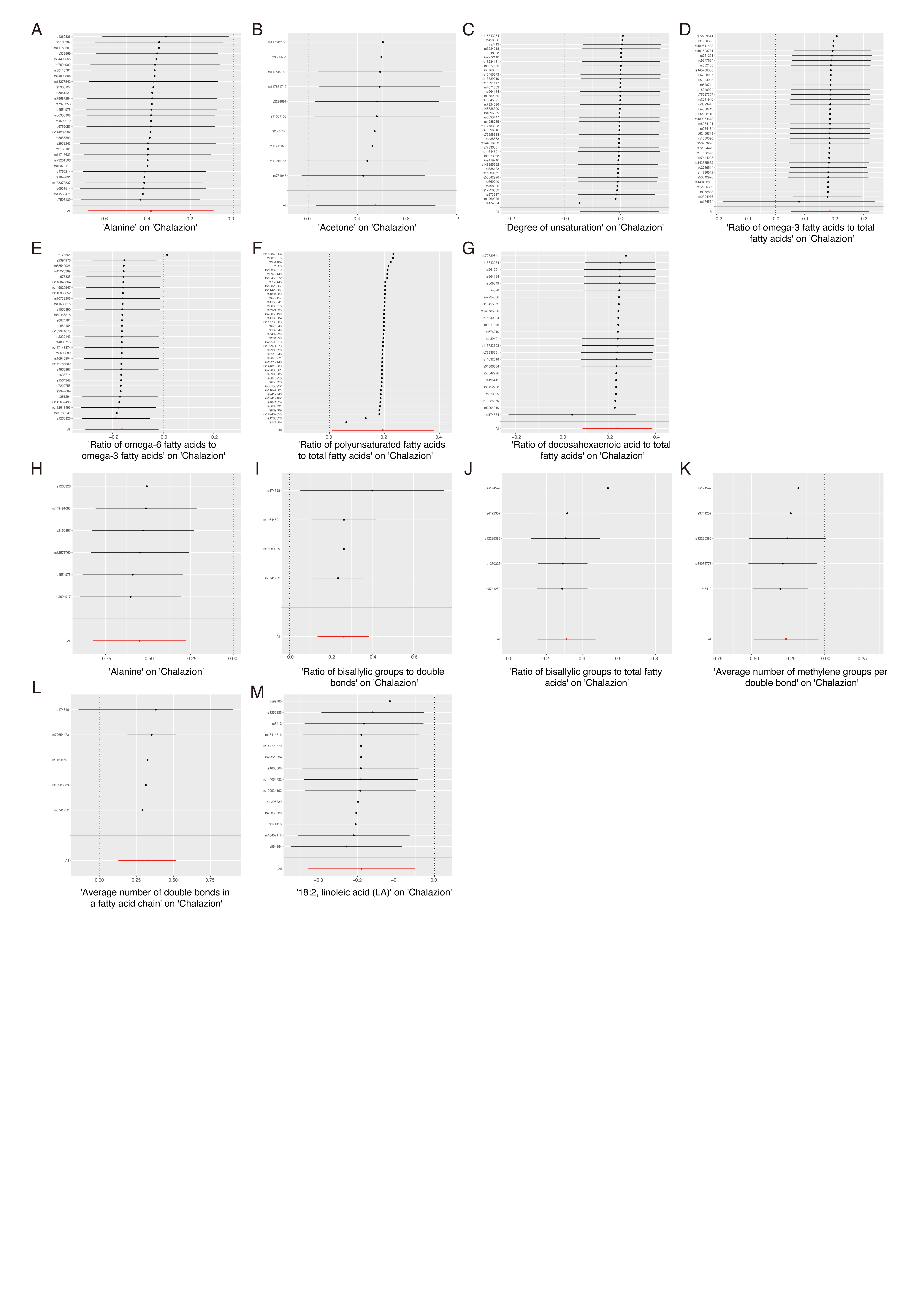

Supplement: Supplementary file 2 [file Image1.TIF]
